# Body areas satisfaction and body mass in adolescents: mediating effects of actual–ideal body weight discrepancies

**DOI:** 10.1007/s40519-019-00722-8

**Published:** 2019-06-07

**Authors:** Karolina Zarychta, Karolina Horodyska, Carina K. Y. Chan

**Affiliations:** 1 Wroclaw Faculty of Psychology, SWPS University of Social Sciences and Humanities in Wroclaw, 30b Ostrowskiego Street, 53-238 Wroclaw, Poland; 2 School of Psychology and Public Health, La Trobe University, Melbourne, Australia

**Keywords:** Body areas satisfaction, Body mass index, Cognitive factors, Actual–ideal body weight discrepancy, Adolescence

## Abstract

**Purpose:**

This study aims at investigating prospective associations between body areas satisfaction (BAS), actual (objectively measured)–ideal body weight discrepancy, actual (self-reported)–ideal body weight discrepancy and BMI among adolescents from the general population.

**Methods:**

Data were collected at three measurement points: baseline (T1), 2-month follow-up (T2), 13-month follow-up (T3) among 1011 adolescents (59.3% girls) aged 13–19 years (*M* = 16.30, SD = 0.82) with BMIs ranging from 15.20 to 38.78 (*M* = 20.01, SD = 3.33). Adolescents completed questionnaires regarding BAS (T1), actual and ideal body weight (T2). Body weight and height were measured objectively (T1 and T3).

**Results:**

Adolescents satisfied with most areas of their bodies had lower levels of actual (objectively measured)–ideal body weight discrepancy, which in turn predicted higher BMI, while lower levels of actual (self-reported)–ideal body weight discrepancy predicted lower BMI. No moderating effect of gender was found.

**Conclusions:**

Actual–ideal weight discrepancies may operate in complex manner prompting opposite effects on BMI.

**Level of evidence:**

Level III, longitudinal study without control group.

## Introduction

Excessive body weight as well as underweight among adolescents continues to be frequently discussed and researched topic [1]. Healthy body weight reduces the risk of serious short- and long-term health problems such as diabetes, cardiovascular diseases, and hypertension [2]. Moreover, underweight, overweight and obesity can affect adolescents’ mental health being associated with clinical and subclinical symptoms of eating disorders (EDs) [3, 4]. At the same time, World Health Organization (WHO) [5] estimates that up to 13.7% adolescences worldwide are underweight, and about 21.7% are either overweight or obese (applying the International Obesity Task Force thresholds [6]). WHO [7] suggests that healthy diet and physical activity are key factors for obtaining or maintaining healthy body weight. However, cognitive theories [8, 9] indicate that cognitions precede the behaviors (i.e., related to diet and physical activity). This cognition may include the ways individuals perceive their body appearance, weight and shape. Therefore, the identification of cognitive factors promoting healthy body weight can help in the prevention of underweight and excessive weight consequences in the future.

One of such factors is body image, a multidimensional construct accounting for cognitive, emotional, and behavioral factors regarding one’s appearance, body weight and shape [8]. Both cognitive (i.e., body weight discrepancies) and emotional (i.e., body satisfaction) dimensions of body image were found to be significant determinants of body mass index (BMI) [10]. Body satisfaction is assumed to be the lowest during the adolescence when body image significantly changes enlarging the discrepancy between adolescent’s actual and ideal body weight [11]. The body discrepancy theory [12] was adapted from the self-discrepancy theory to explain body weight or BMI [13]. The adapted version focus on discrepancies between actual and ideal body weight with higher levels of actual–ideal body weight discrepancies acting as a predictor of BMI alternations since individuals may try to reduce discrepancies by introducing healthy or unhealthy changes in their physical activity levels and nutrition behaviors [9]. Therefore, body satisfaction and actual–ideal body weight discrepancies may act as emotional and cognitive determinants of BMI. Even though body image is a well-defined multidimensional construct, researchers seldom test more than just one dimension in the model. It may be due to wrongly equating actual–ideal body weight discrepancies which represent cognitive process with body satisfaction which represents emotional state, that both refer to body appearance, weight and shape [8]. In the present study, both emotional and cognitive dimensions of body image are taken into account.

Previous research [9, 14, 15] on body weight perceptions have most often used self-reported discrepancies since they are easier to asses than objectively measured discrepancies. Indeed, some studies indicated that the objective reality (actual body weight) is a weaker correlate of future BMI than the subjective perception of that reality (perceived body weight) [15, 16]. However, other studies showed that the recognition of objectively measured body weight as unsatisfactory [17] as well as subjectively perceiving body weight as unsatisfactory [18] are both predictors of BMI alterations since they are associated with higher actual–ideal body weight discrepancies leading to favorable or unfavorable health behavior changes [19]. On the other hand, adolescents who are satisfied with their bodies, with lower actual–ideal body weight discrepancies can be less motivated to change their health behaviors [20]. There is a sparse number of studies using both self-reported and objectively measured body weight discrepancies [15] with no studies testing the mediating role of both of them between body areas satisfaction and adolescents’ BMI. The present study aimed to fill this gap.

Both body satisfaction and actual–ideal body weight discrepancies are assumed to be highly associated with the gender, as suggested by the sociocultural model [21] which suggest that women are less satisfied with their bodies than men [22–24]. Such gender differences were also highlighted to be evolutionary in nature [25]. However, body satisfaction is an important issue for both genders with women, and overweight or obese men strive to lose weight, and underweight men to gain weight [23, 24]. It is also known that genders differ also in terms of body parts preoccupation with women strive to have hourglass figure and men to have V-shaped figure [23]. This kind of discrete body areas satisfaction (BAS) constitutes an overall body satisfaction measure in many studies since they were found to be highly intercorrelated [26]. Gender-specific differences led researchers to test body satisfaction in separate men and women samples [27]. However, to assess the actual effects of gender, it should be included into analyses as a moderator. Thus, the present study aimed at testing the associations between study variables with gender included as a moderator in respective analyses.

There are many cross-sectional studies and only a few longitudinal studies that confirmed the associations between body satisfaction, actual–ideal body weight discrepancies and adolescents’ BMI [20, 28]. However, to understand temporal effects of BAS, actual–ideal body weight discrepancies and BMI, variables should be measured at separate time points [29]. Moreover, most of the previous research have tested direct associations between above-mentioned variables [27] or referred only to clinical or excessive body weight samples of adolescents [30]. Yet, body satisfaction and its associations with actual–ideal body weight discrepancies, and BMI are important for adolescents in general since the consequences of underweight and excessive weight are similar in both genders [5, 6]. The relation between BAS and BMI as being mediated by both indices of actual (self-reported and objectively measured)–ideal body weight discrepancy was not investigated so far. It might be due to the frequent usage of self-reported measures of BMI in previous research [14]. Summing up, previous studies usually applied cross-sectional design, relied only on self-report measures, and did not include gender as a moderator or a covariate. The present study addressed these issues by applying prospective design (with variables being measured at three measurement points: at the baseline, 2- and 13-month follow-ups) to establish temporal precedence, relying on self-report and objective measures, including gender as a moderator or a covariant in respective analyses, and investigating direct and indirect associations between study variables among adolescents with a wide range of BMI.

In conclusion, this study provides an insight into prospective associations between body areas satisfaction (Time 1; T1), two indices of actual–ideal body weight discrepancies [actual (objectively measured)–ideal body weight discrepancy and actual (self-reported)–ideal body weight discrepancy] (Time 2; T2), and adolescents’ BMI z-scores (Time 3; T3). In particular, it was hypothesized that:

1. the association of body areas satisfaction (T1) and BMI z-scores (T3) would be mediated by actual (objectively measured)–ideal body weight discrepancy (T2) and actual (self-reported)–ideal body weight discrepancy (T2); (Hypothesis 1; H1);
2. the associations of body areas satisfaction (T1) with actual–ideal body weight discrepancies (T2), and actual—ideal body weight discrepancies (T2) and BMI z-scores (T3) would be moderated by participants’ gender (Hypothesis 2; H2).

Since BMI is changing rapidly during the adolescence, the effect of time was taken into account as a main confounder of the dependent variable. Therefore, both hypotheses were tested controlling for the effects of BMI z-scores (T1), age and gender (where appropriate) on the dependent variable.

## Methods

### Participants

At T1 (T1; baseline), 1011 adolescents participated in the study. At Time 2 (T2; 2-month follow-up), 992 adolescents continued the study and 938 individuals provided their data at Time 3 (T3; 13-month follow-up). All participants were Caucasian (98% of Poland’s population is White; see Demographic Yearbook of Poland [31]). Table 1 provides information about participants’ age, gender, and BMI.

**Table 1:** The descriptive characteristics of the sample

	Time 1	Time 2	Time 3
*N*	1011	992	938
Age	13–19 (*M* = 16.30, SD = 0.82)	13–19 (*M* = 16.57, SD = 0.87)	14–19 (*M* = 17.16, SD = 0.78)
Female (%)	59.3%	56.2%	55.8%
BMI	15.20–38.78 (*M* = 20.01, SD = 3.33)	15.61–38.67 (*M* = 21.90, SD = 3.27)	15.42–35.56 (*M* = 21.19, SD = 2.91)
Adolescents with underweight (%)	0.6%	0.9%	0.7%
Adolescents with normal weight (%)	77.7%	80.2%	85.2%
Adolescents with overweight (%)	17.5%	14.6%	10.3%
Adolescents with obesity (%)	4.2%	4.3%	1.9%

The total attrition rate was 7.22% and was linked usually to finishing, changing or dropping out of school by participants. Any missing data were imputed, including data missing due to the longitudinal dropout and data missing for specific items at any measurement points. Thus, data collected from *N* = 1011 adolescents (59.3% girls) aged 13–19 years (*M* = 16.30, SD = 0.82) with BMIs ranging from 15.20 to 38.78 (*M* = 20.01, SD = 3.33) were included in the analyses.

### Procedure

Participants were enrolled in 16 middle and high public schools. All respondents lived with their parents (98.9%) or other legal guardians (1.1%) at all measurement points. Participants and parents of those younger than 18 years old provided informed consent prior to the data collection. Informed consent obtained from the parent and the participants, and being at least 13 and not more than 19 years old at T1 were the only inclusions criteria. There were no exclusion criteria. Individuals were informed about the objectives and the procedure of the study, and were assigned personal codes to secure anonymity and identification across the measurement points. Collection of adolescents’ data took place at schools. Participants provided their data in a questionnaire referring to their body areas satisfaction, and actual and ideal body weight. This procedure was performed at all three measurement points. The intervals between the assessment points were chosen to account for the short-term and long-term associations between the study variables. After completing the questionnaires, at T1 and T3, adolescents’ body weight and height were measured objectively and individually by researchers in another room or in the school nurse’s office. Researchers (three women with a MA degree in clinical psychology who participated in a data collection training) were available for consultations after study completion, if desired. Also, they returned to schools 3–5 times across a 3-week period after T2 and T3 to reduce attrition.

All the procedures performed in the study were in accordance with the ethical standards of the Institutional Review Board and Ethics Committee at the SWPS University of Social Sciences and Humanities in Wroclaw, Poland and with the 1975 Helsinki Declaration and its later amendments.

### Materials

Means, standard deviations, and results of variance analyses between boys and girls are presented in Table 2. Validity of the measures used in the study was confirmed elsewhere [32, 33].

**Table 2:** Descriptive statistics, between-group differences, and correlations between the study variables at T1 and T2 (*N* = 1011)

		Differences between boys and girls: F	M (SD) for boys/M (SD) for girls	Total sample: M (SD)	2	3	4	5	6	7
1	T1 body areas satisfaction	49.72***	3.45 (0.69)/3.13 (0.72)	3.26 (0.73)	− 0.23***	− 0.25***	− 0.15***	− 0.08*	0.05	0.22***
2	T2 actual (objectively measured)–ideal body weight discrepancy	168.33***	− 1.15 (9.02)/5.60 (7.45)	3.09 (9.33)		0.92***	0.57***	0.43***	− 0.02	− 0.39***
3	T2 actual (self-reported)–ideal body weight discrepancy	185.89***	− 1.35 (9.32)/6.13 (8.03)	2.85 (8.77)			0.61***	0.45***	− 0.02	− 0.38***
4	T1 BMI z-scores	7.62**	0.39 (0.88)/0.22 (1.03)	0.29 (0.97)				0.85***	− 0.03	0.09**
5	T3 BMI z-scores	53.71***	0.15 (0.81)/− 0.32 (1.05)	− 0.13 (0.99)					0.01	0.23***
6	T1 age	0.001	16.30 (0.69)/16.30 (0.82)	16.30 (0.82)						0.01
7	Gender									

*T1* Time 1, baseline, *T2* Time 2, 2-month follow-up, *T3* Time 3, 13-month follow-up, *BMI* body mass index; gender was coded—1 for boys and + 1 for girls

***p < 0.001; **p < 0.01; *p < 0.05; †*p* < 0.1

#### Body areas satisfaction (BAS) (T1)

Body areas satisfaction was measured with The Multidimensional Body-Self Relations Questionnaire’s Body Areas Satisfaction Subscale (MBSRQ) [34]. The subscale evaluates feelings of physical attractiveness or unattractiveness with discrete aspects of one’s appearance. Adolescents were asked to indicate how dissatisfied or satisfied they are with nine following areas of their body: face (facial features, complexion), hair (color, thickness, texture), lower torso (buttocks, hips, thighs, legs), mid torso (waist, stomach), upper torso (chest or breast, shoulders, arms), muscle tone, weight, height, and overall appearance. The responses ranged from one (*very dissatisfied*) to five (*very satisfied*). High scores relate to individuals generally content with most areas of their body or its size and low scores relate to individuals unhappy with several areas of their body or its size. Cronbach’s *α* was 0.82.

#### Body weight and height (T1 and T3)

Standard medically approved telescopic height-measuring rods and floor scales (scale type: BF-100 or BF-25) were used to obtain biometric measures. Age- and gender-specific BMI *z*-scores and percentiles were assessed with WHO AnthroPlus macro [35], which was created based on the WHO growth Ref. [36] for children and adolescents. Thus, the BMI indicator of each participant accounts for their age and gender. BMI *z*-scores were used as independent variables in all analyses.

#### Actual (objectively measured or self-reported)–ideal body weight discrepancies (T2)

To assess adolescents’ ideal body weight, they were asked to provide an answer to a following question: “How much would you like to weight? (in kilograms)”. Actual (self-reported) body weight was measured with the weight in kilograms provided by adolescents prior the measurement of their actual (objectively measured) body weight assessed using a certified body weight scale (as described in the point above).

The actual (objectively measured)–ideal body weight discrepancy was obtained by subtracting the actual body weight (objectively measured) from the ideal body weight. The actual (self-reported)–ideal body weight discrepancy was assessed by subtracting the actual body weight (self-reported) from the ideal body weight. Similar procedure of assessing body weight discrepancies was applied in the previous studies [14].

### Data analysis

**Fig. 1:**
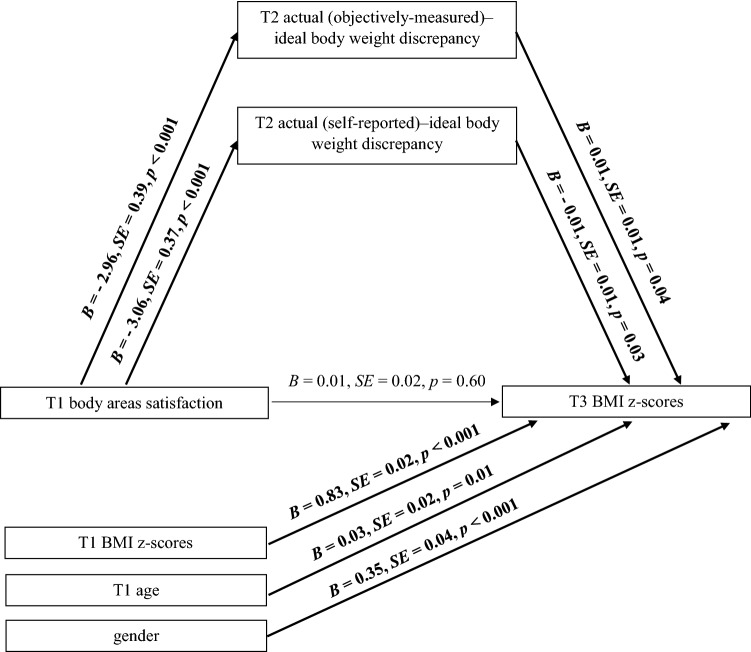
Effects of body areas satisfaction on adolescents’ BMI z-scores through actual (objectively measured)–ideal weight discrepancy and actual (self-reported)–ideal weight discrepancy. *T1* Time 1, baseline, *T2* Time 2, 2-month follow-up, *T3* Time 3, 13-month follow-up. Paths marked in bold represent significant associations

Data were analyzed using SPSS version 24 and PROCESS macro-version 2.16.3 with 10,000 bootstraps [37]. A multiple mediation analysis (Model 4) was performed to test the associations between T1 body areas satisfaction (independent variable; IV), both indices of T2 actual–ideal body weight discrepancies (mediators), and adolescents’ T3 BMI z-scores (dependent variable; DV) accounting for the covariates (T1 BMI z-scores, age and gender). Further, moderated multiple mediation analysis (Model 59) was conducted to test if the associations between (1) the IVs and the DV, (2) the IVs and the mediators, and (3) the mediators and the DV are moderated by gender accounting for covariates (T1 BMI *z*-scores and age). Two types of coefficients present the results of the analyses: (1) a regression coefficient for each parameter (see Fig. 1), and (2) the indirect effect coefficient (*B*) for each indirect pathway between the IV (T1 BAS) and the DV (T3 BMI *z*-scores), accounting for respective mediators and covariates (see Table 3).

**Table 3:** Effects of body areas satisfaction on adolescents’ BMI *z*-scores through actual–ideal weight discrepancies

Indirect effect pathways	*B*	*SE*	BC 95% CI	
			Lower	Higher
Testing the effect of body areas satisfaction on adolescents’ BMI z-scores through body weight discrepancies (H1)				
Model 1				
T1 body areas satisfaction → T2 actual (objectively measured)–ideal body weight discrepancy → T3 BMI z-scores	0.03	0.01	− 0.06	− 0.01
T1 body areas satisfaction → T2 actual (self-reported)–ideal body weight discrepancy → T3 BMI z-scores	− 0.02	0.01	− 0.05	− 0.01
	Moderated mediation index	*SE*	BC 95% CI	
			Lower	Higher
Testing the moderating role of gender (H2)				
Model 2				
Gender moderating T1 body areas satisfaction → T3 BMI *z*-scores	0.07	0.07	− 0.02	0.16
Gender moderating T1 body areas satisfaction → T2 actual (objectively measured)–ideal body weight discrepancy	1.67	0.77	− 0.16	3.19
Gender moderating T1 body areas satisfaction → T2 actual (self-reported)–ideal body weight discrepancy	1.68	0.73	− 0.25	3.11
Gender moderating T2 actual (objectively measured)–ideal body weight discrepancy → T3 BMI *z*-scores	0.01	0.01	− 0.01	0.03
Gender moderating T2 Actual (self-reported)–ideal body weight discrepancy → T3 BMI *z*-scores	− 0.02	0.01	− 0.04	0.01

Values of indirect effect coefficient (*B*) presented in bold are significant. Each bootstrap was based on 10,000 repetitions. Bias-corrected (BC) confidence intervals (CI) that do not include zero indicate a significant indirect effect

*T1* Time 1, baseline, *T2* Time 2, 2-month follow-up, *T3* Time 3, 13-month follow-up, *BMI* body mass index

In this study, the IV was BAS (T1); the DV was BMI *z*-score measured at T3; the mediators were actual (objectively measured)–ideal body weight discrepancy (T2) and actual (self-reported)–ideal body weight discrepancy (T2); moderator was gender (T1) coded as—1 for boys and + 1 for girls. To establish temporal precedence [35], the IV, the mediators, and the DV in the respective analyses were measured at different time points (T1, T2 and T3). Missing data were imputed with multiple imputation method. A total of 4.3% of the completers’ data were missing. The attrition analysis is presented below.

## Results

### Preliminary analyses

#### Attrition analysis

There were no differences between completers and dropouts in terms of both indices of actual–ideal body weight discrepancies, BAS and BMI *z*-scores, all *F*s < 1.11, ps > 0.19, or gender, *χ*^2^ (1) = 0.10, *p* = 0.76. Completers and those who dropped out at T2 or T3 differed in age, *F* (1, 1010) = 34.33, *p* < 0.0001 with dropouts being slightly older (*M* = 17.30, SD = 0.60) than completers [*M* = 17.01, SD = 0.92, Cohen’s *d* = 0.38 (95% CI 0.33–0.42)].

#### Correlation analyses and comparisons between genders

Correlations analyses between all variables for the study sample (*N* = 1011) are presented in Table 2. BAS (T1) were weakly and negatively associated with both indices of actual–ideal body weight discrepancies (T2) and adolescents’ BMI *z*-scores (T1 and T3), but weakly and positively with gender. Both indices of actual–ideal body weight discrepancies (T2) were strongly and positively related to each other, moderately and positively to BMI *z*-scores (T1 and T3), and moderately and negatively to gender. There were strong positive correlations between BMI *z*-scores measured at T1 and T3, and very weak or weak positive correlations between both BMI z-scores (T1 and T3) and adolescents’ gender.

Also, differences in study variables were tested for both genders. Significant differences were found between boys and girls in terms of BAS (T1), both indices of actual–ideal body weight discrepancies (T2) and BMI *z*-scores (T1 and T3) with boys having higher BMI *z*-scores, but also being more content with most parts of their bodies and perceiving their body weight more accurately since both indices of actual–ideal body weight discrepancies were lower in boys, all *F*s > 168.33, ps < 0.05 (see Table 2).

### Indirect effect of BAS on BMI *z*-scores: testing the mediating role of actual–ideal body weight discrepancies

Model 1 (see Table 3) was designed to test the indirect effect of *BAS* (T1) (IV) on *BMI z*-*scores* (T3) (DV) through *actual (objectively measured)–ideal body weight discrepancy* (T2) and *actual (self*-*reported)*–*ideal body weight discrepancy* (T2) (see Fig. 1). Analysis was performed controlling for adolescents’ gender, age and BMI *z*-scores at T1.

The results indicated significant indirect effect of *BAS* (T1) on *BMI z*-*scores* (T3) through *actual (objectively measured)*–*ideal body weight discrepancy* at T2 [*R*^*2*^= 0.05, *F* (1, 1008) = 56.54, *p* < 0.001] and through *actual (self*-*reported)*–*ideal body weight discrepancy* at T2 [*R*^*2*^ = 0.06, *F* (2, 336) = 68.96, *p* < 0.001] (see Model 1 in Table 3). We have also found direct relations between IV (T1), mediators (T2) and DV (T3). Adolescents who were generally content with most areas of their body (T1) also had lower discrepancy between their actual (objectively measured) and ideal body weight (T2) which in turn predicted higher BMI *z*-scores at T3. A different pattern of findings was observed for actual (self-reported)–ideal body weight discrepancy: adolescent who were generally content with most areas of their body (T1) also had lower discrepancy between their actual (self-reported) and ideal body weight (T2) which in turn predicted lower BMI *z*-scores at T3.

The model including only one mediator explained 5% [when actual (objectively measured)–ideal body weight discrepancy was considered] or 6% [when actual (self-reported)–ideal body weight discrepancy was considered] of the variance. The whole model, including IV (BAS), both mediators (two types of actual–ideal body weight discrepancies) and covariants (adolescents’ gender, age and BMI *z*-scores at T1) explained 74% of the variance, *R*^*2*^ = 0.74, *F* (6, 1003) = 485.03, *p* < 0.001.

### Testing the moderating role of gender

Model 2 was designed to test whether *gender* moderated the relations between (1) *BAS* (T1) (IV) and both indices of *actual*–*ideal body weight discrepancies* (T2) (mediators), and (2) between mediators (T2) and *BMI z*-*scores* (T3) (DV). It was measured with moderated multiple mediation analysis, controlling for adolescents’ BMI *z*-scores (T1) and age.

The results of moderated multiple mediation analysis (see Model 2 in Table 3) indicated no significant effects of *gender* on any of above-mentioned associations. Thus, the outcomes were the same for girls and boys.

## Discussion

Present study provides new evidence for the prospective associations between body areas satisfaction, two indices of actual–ideal body weight discrepancies [actual (objectively measured)–ideal body weight discrepancy and actual (self-reported)–ideal body weight discrepancy] and objectively measured BMI among adolescents from the general population. The results showed that BAS is indirectly associated with adolescents’ BMI *z*-scores through both indices of actual–ideal body weight discrepancies. However, these two cognitive mediators have different effects: adolescents who were satisfied with most areas of their bodies (T1), also had lower levels of actual (objectively measured)–ideal body weight discrepancy (T2), which in turn predicted higher BMI z-scores (T3), while lower levels of actual (self-reported)–ideal body weight discrepancy (T2) predicted lower BMI *z*-scores (T3). No moderating effect of gender was found.

The first hypothesis suggested that BAS may have indirect effects (through two indices of actual–ideal body weight discrepancies) on adolescent’s BMI *z*-scores. This hypothesis has been confirmed. BAS was a predictor of both indices of actual (objectively measured)–ideal body weight discrepancy and actual (self-reported)–ideal body weight discrepancy, and in turn, both indices were predictors of adolescents’ BMI z-scores. Both indices of actual–ideal body weight discrepancies operated as mediators between BAS and BMI *z*-scores. However, it is worth highlighting that these two mediators operate in different ways with actual (objectively measured)–ideal body weight discrepancy predicting higher BMI *z*-scores, and actual (self-reported)–ideal body weight discrepancy predicting lower BMI *z*-scores. Direct associations between BAS and actual–ideal body weight discrepancies [38], and between actual–ideal body weight discrepancies and BMI [39] have been confirmed in the previous studies. However, no studies tested indirect associations between these variables. Yet, some research evidence has suggested that overweight and obese people satisfied with their bodies and with lower actual–ideal body weight discrepancies can be less motivated to introduce healthy or unhealthy changes in their physical activity levels and nutrition behaviors change and lose weight [20]. This is partially congruent with the findings for actual (objectively measured)–ideal body weight discrepancy. The results found for actual (self-reported)–ideal body weight discrepancy were different as it was a predictor of lower BMI *z*-scores. This may be explained by the findings of several studies [10, 15, 16] showing that the subjective perception of the reality (perceived body weight) is a stronger correlate of future BMI than that reality itself (actual body weight). Therefore, even though both indices of actual–ideal body weight discrepancies were lower when adolescents’ had been content with most areas of their bodies, these two may have operated differently on participants’ BMI *z*-scores.

The second hypothesis tested the moderating effect of gender in the associations between BAS, actual–ideal body weight discrepancies and adolescents’ BMI *z*-scores. Based on previous studies, it has been expected that the observed effects will be gender specific, with stronger associations between BAS, actual–ideal body weight discrepancies and BMI *z*-scores among girls compared to boys [24]. This hypothesis was not supported. Preliminary analyses indicated significant gender differences with girls being less satisfied with their bodies and have higher levels of both indices of actual–ideal body weight discrepancies than boys. However, no effect of gender was found in moderated mediation model. Gender was not a moderator in the associations between BAS, actual–ideal body weight discrepancies and adolescents’ BMI *z*-scores. Therefore, these patterns of associations can be seen as gender independent. This finding is partially in line with previous studies [40] indicating that gender is a significant covariant yet not a moderator of the associations between the present study’s variables.

The results of this study allowed for more thorough prospective analysis of the association between BAS, actual–ideal body weight discrepancies and objectively measured BMI *z*-scores than in the previous cross-sectional studies that explored direct associations between variables [38, 39]. Moreover, the findings indicated that these associations seem to be consistent for a large sample of both boy and girl participants and across the range of body weight status.

The present study has several limitations. The first ones are related to study design. Namely, the study variables were not measured at each time point. Therefore, it was not possible to evaluate their change over time. Moreover, even though efforts were made to choose the intervals able to explain the short- and long-term associations between the study variables, the choice of the time points in the study was arbitrary. Further, other variables which were not included in the present study but were found as significant in the explanation of the associations between BAS and BMI, such as: cognitive variables (e.g., perfectionism and self-esteem), emotional variables (e.g., depression, anxiety and emotion regulation), environmental variables (e.g., built physical environment and food accessibility), social variables (e.g., peer, parental, media influence), and behavioral variables (e.g., diet, physical activity and sedentary behavior) [25, 28, 41]. Moreover, it is worth mentioning that body dissatisfaction was previously found to be explained to a large extend by genetic factors [25] that were not controlled in the study. These variables should be included as relevant in the future studies. Also, adolescents were not screened for eating disorders’ symptoms, but they, however, can occur in this group [15] and could have affected the outcomes obtained in the study constituting another factor that could be controlled for in the future. Also, excluding individuals with eating disorders’ diagnosis when conducting similar studies is recommended. Any conclusions should refer to general population of adolescents (thus, including wide range of BMI and manifesting symptoms of many disorders including eating disorders). Generalizations to ethnically diverse populations should be made with caution as the sample analyzed in the present study was ethnically homogeneous (all adolescents were Caucasians). Another limitation may be the wide age range of the sample. However, age was controlled for in all analyses to address this issue. Besides age, other variables indicated previously as being significant confounds of adolescents’ BMI *z*-scores (e.g., baseline BMI *z*-scores) were controlled for, which can explain low beta coefficients obtained in the present study and low explained variance of the model including only mediators. Finally, participants’ responses could be biased by the social desirability.

Concluding, the result of this study confirmed the indirect effects of BAS on BMI *z*-scores through two indices of actual–ideal body weight discrepancies. The findings suggests that factors such as BAS and actual–ideal body weight discrepancies should continue to be use in ED prevention and treatment programs, and in weight management programs. Such programs should target adolescents of both genders and with wide range of BMI *z*-scores, manifesting high levels of one of the two actual–ideal body weight discrepancies [i.e., actual (self-reported)–ideal body weight discrepancy or actual (objectively measured)–ideal body weight discrepancy]. However, it may be more relevant for excessive weight treatment programs to account for actual (objectively measured)–ideal body weight discrepancy since it was found to be a predictor of higher BMI. On the other hand, eating disorder prevention and treatment programs should account rather for actual (self-measured)–ideal body weight discrepancy as it was found to be a predictor of lower BMI. The findings suggest that future research may benefit from testing both indices of actual–ideal body weight discrepancies when assessing underweight or excessive weight risk factors since they both were stimulated by BAS having different consequences on adolescents’ BMI *z*-scores.

## References

[1] Health Behaviour School-aged Children (2017) Adolescent obesity and related behaviours. http://www.hbsc.org/publications/international/ Accessed 3 Jan 2018

[2] Stein CJ, Colditz GA (2004). The epidemic of obesity. J Clin Endocrinol Metab.

[3] Zarychta K, Mullan B, Kruk M, Luszczynska A (2017). A vicious cycle among cognitions and behaviors enhancing risk for eating disorders. BMC Psychiatry.

[4] Zarychta K, Luszczynska A, Scholz U (2014). The association between automatic thoughts about eating, the actual-ideal weight discrepancies, and eating disorders symptoms: a longitudinal study in late adolescence. Eat Weight Disord St.

[5] World Health Organization (2018) Malnutrition. https://www.who.int/news-room/fact-sheets/detail/malnutrition/ Accessed 15 Jan 2018

[6] Cole TJ, Bellizzi MC, Flegal KM, Dietz WH (2000). Establishing a standard definition for child overweight and obesity worldwide. BMJ.

[7] World Health Organization (2012) Population-based approaches to childhood obesity prevention. http://www.who.int/dietphysicalactivity/en/ Accessed 18 Jan 2017

[8] Vartanian LR, Cash T (2012). Self-discrepancy theory and body image. Encyclopedia of body image and human appearance.

[9] Fairburn CG (2008). Cognitive Behavior therapy and eating disorders.

[10] Perkins JM, Perkins HW, Craig DW (2015). Misperception of peer weight norms and its association with overweight and underweight status among adolescents. Prev Sci.

[11] Bratovcic V, Mikic B, Kostovski Z, Teskeredzic A, Tanovic I (2015). Relations between different dimensions of self-perception, self-esteem and body mass index of female students. Int J Morphol.

[12] Higgins ET (1987). Self-discrepancy: a theory relating self and affect. Psychol Rev.

[13] Strauman TJ, Vookles J, Berenstein V, Chaiken S, Higgins ET (1991). Self-discrepancies and vulnerability to body dissatisfaction and disordered eating. J Pers Soc Psychol.

[14] Kärkkäinen U, Mustelin L, Raevuori A, Kaprio J, Keski-Rahkonen A (2016). Ideals versus reality: are weight ideals associated with weight change in the population?. Obesity.

[15] Zarychta K, Luszczynska A, Scholz U (2014). The association between automatic thoughts about eating, the actual-ideal weight discrepancies, and eating disorders symptoms: a longitudinal study in late adolescence. Eat Weight Disord.

[16] Zarychta K, Mullan B, Luszczynska A (2016). It doesn’t matter what they say, it matters how they behave: parental influences on healthy behaviors and changes in body mass among adolescents with overweight and obesity. Appetite.

[17] Yang K, Turk MT, Allison VL, James KA, Chasens E (2014). Body mass index self-perception and weight management behaviors during late adolescence. J Sch Health.

[18] Bodde AE, Beebe TJ, Chen LP, Jenkins S, Perez-Vergara K, Rutten LJF, Ziegenfuss JY (2014). Misperceptions of weight status among adolescents: sociodemographic and behavioral correlates. Patient Relat Outcome Meas.

[19] Ojala K, Tynjala J, Valimaa R, Villberg J, Kannas L (2012). Overweight adolescents’ self-perceived weight and weight control behaviour: hBSC study in Finland 1994–2010. J Obes.

[20] Kuk JL, Ardern CI, Church TS, Hebert JR, Sui X, Blair SN (2009). Ideal weight and weight satisfaction: association with health practices. Am J Epidemiol.

[21] Striegel-Moore R, Bulik CM (2007). Risk factors for eating disorders. Am Psychol.

[22] Feingold A, Mazzella R (1998). Gender differences in body image are increasing. Psychol Sci.

[23] Kostanski M, Fisher A, Gullone E (2004). Current conceptualisation of body image dissatisfaction: have we got it wrong?. J Child Psychol Psychiatry.

[24] Cash TF, Morrow JA, Hrabosky JI, Perry AA (2004). How has body image changed? A cross-sectional investigation of college women and men from 1983 to 2001. J Consult Clin Psychol.

[25] Ferguson CJ, Winegard B, Winegard BM (2011). Who is the fairest one of all? How evolution guides peer and media influences on female body dissatisfaction. Rev Gen Psychol.

[26] Mellor D, Hucker A, Waterhouse M, Binti Mamat NH (2014). A cross-cultural study investigating body features associated with male adolescents’ body dissatisfaction in Australia, China, and Malaysia. Am J Mens Health.

[27] Mäkinen M, Puukko-Viertomies LR, Lindberg N, Siimes MA, Aalberg V (2012). Body dissatisfaction and body mass in girls and boys transitioning from early to mid-adolescence: additional role of self-esteem and eating habits. BMC Psychiatry.

[28] Zarychta K, Chan CKY, Kruk M, Luszczynska A (2018). Body satisfaction and body weight in under- and healthy-weight adolescents: Mediating effects of restrictive dieting, healthy and unhealthy food intake. Eat Weight Disord.

[29] MacKinnon DP (2008). Introduction to statistical mediation analysis.

[30] Sonneville KR, Calzo JP, Horton NJ, Haines J, Austin SB, Field AE (2012). Body satisfaction, weight gain and binge eating among overweight adolescent girls. Int J Obes.

[31] Demographic Yearbook of Poland (2015) Central statistical office for Poland: Warsaw, Poland. https://stat.gov.pl/obszary-tematyczne/roczniki-statystyczne/roczniki-statystyczne/rocznik-demograficzny-2015,3,9.html Accessed 16 Jan 2018

[32] Bowring AL, Peeters A, Freak-Poli R, Lim MSC, Gouillou M, Hellard M (2012). Measuring the accuracy of self-reported height and weight in a community-based sample of young people. BMC Med Res Methodol.

[33] Brytek-Matera A, Rogoza R (2015). Validation of the polish version of the multidimensional body-self relations questionnaire among women. Eat Weight Disord.

[34] Cash TF (2000) Users’ manual for the multidimensional body-self relations questionnaire. http://www.body-images.com Accessed 13 Dec 2017

[35] World Health Organization (2011) WHO Anthro (version 3.2.2, January 2011) and macros. http://www.who.int/growthref/tools/en/ Accessed 5 Dec 2017

[36] De Onis M, Onyango AW, Borghi E, Siyam A, Nishida C, Siekmann J (2007). Development of a WHO growth reference for school-aged children and adolescents. Bull World Health Organ.

[37] Hayes AF (2013). An introduction to mediation, moderation, and conditional process analysis: a regression-based approach.

[38] Argyrides M, Sivitanides M (2017). Body image, self-esteem, media, disordered eating and actual ideal weight discrepancy: findings in Cyprus. Euro J Couns Psychol.

[39] Kakeshita I, Almeida SS (2008). The relationship between body mass index and body image in Brazilian adults. Psychol Neurosci.

[40] Penkal JL, Kurdek LA (2007). Gender and race differences in young adults’ body dissatisfaction. Pers Individ Dif.

[41] Luszczynska A, de Wit JBF, de Vet E (2013). At-home environment, out-of-home environment, snacks and sweetened beverages intake in preadolescence, early and mid-adolescence: the interplay between environment and self-regulation. J Youth Adolesc.

